# Distinct pro-inflammatory responses to pristine and microbially contaminated PET nanoplastics in a human alveolar cell co-culture

**DOI:** 10.3389/ftox.2026.1836922

**Published:** 2026-05-18

**Authors:** Øyvind P. Haugen, Andreas Solberg Sagen, Francesco Barbero, Victor Alcolea-Rodriguez, Raquel Portela, Ivana Fenoglio, Bendik C. Brinchmann, Håkan Wallin, Shan Zienolddiny-Narui, Anani K. Afanou

**Affiliations:** 1 National Institute of Occupational Health (STAMI), Oslo, Norway; 2 Department of Chemistry, University of Torino, Torino, Italy; 3 Instituto de Catalisis y Petroleoquimica (ICP), CSIC, Madrid, Spain

**Keywords:** cell viability, cytokines, microbial components, nanoplastics (NPs), pulmonary inflammation, Toll-like receptors

## Abstract

**Background:**

The small size of nanoplastics (NPs; <1 µm in diameter) facilitates airborne transport, inhalation, and deposition in the lungs, raising significant concerns about potential effects on human health. In occupational settings, such as waste management and recycling facilities, exposure to NPs carrying microbial contaminants may pose an additional health risk to workers. In the present study, we investigated pulmonary cytotoxicity and pro-inflammatory responses after exposure to polyethylene terephthalate nanoplastics (PET-NPs) with or without microbial contaminants.

**Methods:**

PET-NPs were synthesized from a post-consumer juice bottle (PET b001) and commodity PET pellets (PET c000). The presence of microbial contaminants was assessed via receptor activation in HEK293 Toll-like receptor (TLR) reporter cells expressing TLR2 or TLR4. Co-cultures of human alveolar epithelial cells (A549) and monocyte-derived macrophages (dTHP-1) were exposed to PET-NPs (0, 10 or 100 μg/mL) that tested either negative or positive for TLR2 and TLR4 activation. After 24 h, cell viability was measured, and cytokine responses were quantified at both mRNA and protein levels.

**Results:**

PET b001 activated TLR2 and TLR4, indicating the presence of biologically active microbial components, whereas PET c000 showed no activation. In A549/dTHP-1 co-cultures, PET b001 (10 and 100 μg/mL) significantly increased IL-1B, IL-6, IL-8, and TNF mRNA levels and IL-6 and IL-8 protein secretion. In comparison, PET c000 selectively increased IL-8 mRNA levels and protein secretion, and only at the highest tested concentration (100 μg/mL). No changes in cell viability were observed for either particle type.

**Conclusion:**

We found that the pro-inflammatory responses to PET-NPs are largely mediated by associated microbial components rather than the polymer itself, highlighting the importance of accounting for environmental context when evaluating their health risks. No evidence of cytotoxicity was observed, as cell viability remained unchanged. Our results further emphasize the need to assess microbial contamination prior to toxicity testing and point to potential occupational health risks in plastic waste and recycling environments.

## Graphical Abstract

**Figure:**
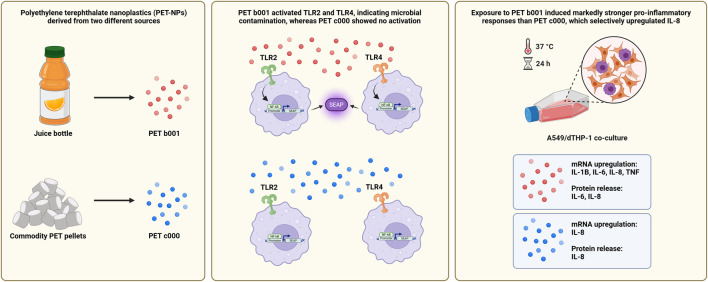
Created in BioRender. Haugen, Ø. (2026) https://BioRender.com/dxeu74h.

## 1 Introduction

Micro- and nanoplastics (MNPs) originate from the fragmentation of larger plastic items or during industrial manufacturing and processing of plastic. They are classified by size, with microplastics (MPs) having diameters less than 5 mm and nanoplastics (NPs) typically defined as particles smaller than 1 µm (Gigault et al., 2018). Their widespread presence in the environment from plastic pollution has led to health concerns, especially after reported findings in human tissues and organs (Anik et al., 2025; Singh and Tiwari, 2025; Ramsperger et al., 2023). However, whether MNPs are toxic and thus pose a risk to human health remains to be determined, although an increasing number of studies are reporting adverse effects (Li and Liu, 2024). A recent hazard-based analysis identified over 4,200 chemicals of concern in plastics, reinforcing concerns about their impacts on human health (Monclús et al., 2025). With the outbreak of the COVID-19 pandemic, potential health risks from inhalation of MNPs gained increased attention as people started using disposable polypropylene masks (Bhangare et al., 2023; Li et al., 2021; Guo et al., 2023).

Inhalation is the main exposure route of concern from an occupational health perspective. It is well documented that occupational exposure to airborne MPs in the synthetic textile, flocking, and polyvinyl chloride (PVC) industries can cause respiratory symptoms and disease (Prata, 2018). In contrast, little is known about the potential health effects from occupational exposure to airborne NPs as exposure data is missing. Inhalation of particles smaller than 100 nm in diameter is of particular concern due to their propensity to deposit in the alveolar region of the lungs, where they may persist for extended periods (Oberdörster, 2000). Adverse effects from toxicity studies using NPs appear to be more pronounced than those of MPs (Foss, 2025). This is likely because particle toxicity is more closely linked to surface area and particle number rather than mass, which makes them more bioactive (Oberdörster et al., 2005). Current analytical methods remain limited in their ability to detect and quantify NPs, and there is a lack of comprehensive international guidance or standardized protocols for their measurement (Cai et al., 2021; Ivleva, 2021). However, particle emissions from plastic processing can be substantial, reaching concentrations of ∼10^10^ particles/m^3^ for 10–469 nm particles during injection molding and ∼10^10^–10^12^ particles/m^3^ for 10–420 nm particles generated during plastic shredding (Swinnerton et al., 2024; Zheng et al., 2025). Workers engaged in the manufacture and processing of plastics may therefore be exposed to high levels of airborne NPs.

Plastic waste for recycling is likely to be contaminated with microorganisms, and elevated levels of endotoxin and other microbial components have been detected in air samples from waste management and recycling facilities (Hansen et al., 2023; Park et al., 2011; Eriksen et al., 2023). In these workplaces, NPs may function as carriers of microbial components given their high surface area-to-mass ratio and hydrophobic nature, although this warrants further investigation. Of note, microbial components like endotoxins and beta glucans have been associated with respiratory symptoms like allergy and asthma in workers (Eduard et al., 2012). To our knowledge, the pulmonary health effects of co-exposure to NPs and microbial contaminants have not been investigated, although such studies are warranted given the continued rise in global plastic production and waste processing (Geyer and Letcher, 2020; Dokl et al., 2024).

We therefore aimed to investigate cell viability and pro-inflammatory responses to polyethylene terephthalate nanoplastics (PET-NPs), comparing microbially contaminated particles with contaminant-free counterparts. To achieve this, PET-NPs synthetized from commodity pellets or a post-consumer juice bottle were screened for microbial contamination using Toll-like receptor (TLR) reporter cells. Co-cultures of alveolar epithelial cells (A549) and monocyte-derived macrophages (dTHP-1) were then exposed for 24 h to either microbially contaminated or pristine PET-NPs, and cell viability and cytokine expression were subsequently assessed.

## 2 Materials and methods

### 2.1 Bottom-up synthetized polyethylene terephthalate nanoplastics

Two different PET-NP water suspension samples, PET b001 and PET c000, were prepared within the framework of PlasticsFatE (EU Horizon 2020 project n. 965,367) by dissolution of PET pieces in hexafluoroisopropanol and subsequent precipitation of nanosized PET nanospheres in Milli-Q water (Robles-Martín et al., 2023; Altmann et al., 2025). The solvent and excess water were removed via rotary evaporation until the final desired concentration was obtained. The starting material for PET b001 was a post-consumer juice bottle (of the brand Granini, purchased in Spain), whereas the starting material for PET c000 was PET pellets intended for industrial use (RAMAPET N180, Indorama Ventures, Thailand). The obtained nanoplastics are spherical, retain the polymer properties, and are predominantly amorphous (Altmann et al., 2025). They were considered as relevant particles for simulating PET nanoplastics with or without microbial ligands.

### 2.2 Particle characterization by dynamic light scattering (DLS)

The size distribution by intensity, the mean hydrodynamic diameter (Z-average) and polydispersity index (PdI) of PET-NPs in cell culture medium (RPMI 1640 supplemented with 10% FBS, 100 U/mL penicillin, and 100 μg/mL streptomycin) were measured by dynamic light scattering (DLS). PET-NP stock solutions were vortexed for 15 s and diluted to a final concentration of 100 μg/mL in cell culture medium. An equal volume of endotoxin-free water was added to cell culture medium containing no PET-NPs. One milliliter of either solution was then transferred to a polystyrene cuvette (#67.754, Sarstedt, Germany) with lid and analyzed on a Zetasizer Nano ZS instrument (Malvern Panalytical, UK) with a light source wavelength of 632.8 nm and the following settings: measurement angle = 173°, temperature = 37 C°, and equilibration time = 120 s. The dispersant medium, RPMI 1640 supplemented with 10% FBS, was assigned a viscosity of 0.958 cP (Poon, 2022) and a refractive index of 1.330. Six consecutive measurements were taken prior to and after 24 h incubation at 37 °C in a humidified 5% CO_2_ incubator to mimic the culture conditions of exposed cells.

### 2.3 Particle characterization by field-emission scanning electron microscopy (FE-SEM)

Field emission scanning electron microscopy (FE-SEM) was used to visualize the particle size and morphology of both PET-NP samples. Each sample was pipetted dropwise onto separate 25 mm nuclepore polycarbonate membrane filters (Cytiva, Marlborough, USA) and left to air-dry under sterile conditions. The filters were subsequently mounted on 25 mm aluminum stubs (Agar Scientific Ltd., Stansted Essex, UK) using double-sided adhesive carbon discs and coated with 10 nm platinum in a Cressington 200HR sputter coater (Cressington Scientific Instruments Ltd., Watford, UK). Micrographs of coated samples were taken with an SU6600 FE-SEM (Hitachi, Ibaraki-Ken, Japan) operated in secondary electron (SE) mode with acceleration voltage 15.0 kV, extraction voltage 1.8 kV, and a working distance between 9.5 and 10.5 mm.

### 2.4 Cell lines

Human leukemia monocytes (THP-1) and alveolar type II epithelial cells (A549) were purchased from ATCC, USA, and HEK-Blue™ hTLR reporter cells were purchased from Invivogen, France. All the cell lines were cultured in their respective culture medium with supplements (Table 1) and maintained at 37 °C in a humidified 5% CO_2_ incubator. The cell cultures were tested and confirmed negative for *mycoplasma* contamination prior to and after experiments using the MycoStrip™ *Mycoplasma* Detection Kit (#rep-mys, Invivogen, France) in accordance with the manufacturer’s protocol.

**TABLE 1:** Cell culture media and supplements used for mono- and co-cultures. DMEM, Dulbecco’s Modified Eagle Medium (low glucose #21885, high glucose #31966, Gibco, Scotland); RPMI 1640, Roswell Park Memorial Institute 1640 (ATCC modification, #A10491, Gibco, Scotland); FBS, Fetal Bovine Serum (ultra-low endotoxin, #S1860, Biowest, France).

Cell line	Culture medium	Serum	Supplements
A549	DMEM, low glucose (1 g/L)	10% (v/v) FBS, ultra-low endotoxin	​
THP-1	RPMI 1640 (ATCC modification)	10% (v/v) FBS, ultra-low endotoxin	​
A549/dTHP-1	RPMI 1640 (ATCC modification)	10% (v/v) FBS, ultra-low endotoxin	100 U/mL penicillin 100 μg/mL streptomycin
HEK-Blue™ hTLR2 HEK-Blue™ hTLR4	DMEM, high glucose (4.5 g/L)	10% (v/v) FBS, ultra-low endotoxin	100 U/mL penicillin 100 μg/mL streptomycin 100 μg/mL Normocin™ HEK-Blue™ Selection antibiotics (1:250 dilution)
HEK-Blue™ Null1	DMEM, high glucose (4.5 g/L)	10% (v/v) FBS, ultra-low endotoxin	100 U/mL penicillin 100 μg/mL streptomycin 100 μg/mL Normocin™ 100 μg/mL Zeocin™

### 2.5 Assessment of PET nanoplastic sterility using Toll-like receptor reporter HEK cells

The potential presence of microbial contaminants was assessed using HEK-Blue hTLR™ reporter cells expressing TLR2 or TLR4 by measuring the levels of NF-κB/AP-1 inducible secreted embryonic alkaline phosphatase (SEAP) in response to particle exposure. The procedure was carried out as previously described by Eriksen et al. (2022), with slight modifications. In brief, 180 μL of HEK-Blue™ hTLR2, HEK-Blue™ hTLR4, or HEK-Blue™ Null1 cells (2.8 × 10^5^ cells/mL) were seeded in triplicates of a 96-well plate and incubated for 24 h. The cells were then treated with 20 µL aliquots of either PET b001 or PET c000 at concentrations ranging from 100 μg/mL to 3.125 μg/mL, prepared by two-fold serial dilution in endotoxin-free water (#J65589, Thermo Scientific, USA). A similar volume of endotoxin-free water served as the negative control. Purified lipoteichoic acid (LTA) from *S. aureus* (#tlrl-psltam, Invivogen, France) and ultra-purified lipopolysaccharide (LPS) from *E. coli* (#tlrl-3pelps, Invivogen, France) at a final concentration of 100 ng/mL were used as positive controls for activation of TLR2 and TLR4, respectively. The cells were then left to incubate for another 24 h before transferring 20 μL of the supernatant to a new 96-well plate and adding 180 μL of QUANTI-Blue™ Solution (#rep-qbs, Invivogen, France) to each of the wells. Following a 3-h incubation at 37 °C, the color development was measured at an absorbance of 649 nm using a BioTek Synergy Neo2 multi-mode microplate reader with Gen four software (BioTek Instruments, USA). The levels of SEAP were determined by calculating the fold change relative to the negative control.

### 2.6 Differentiation of THP-1 monocytes into dTHP-1 macrophages

THP-1 cells (8.0 × 10^5^) were centrifuged at 200 *g* for 5 min and resuspended in 10 mL of fresh ATCC-modified RPMI containing phorbol 12-myristate 13-acetate (PMA) (#P8139, Sigma-Aldrich, USA) at a final concentration of 50 ng/mL (∼80 nM). The cell suspension was then transferred to a TC-treated T75 flask and left to differentiate into adherent macrophage-like cells (dTHP-1) for 72 h in a 37 °C humidified 5% CO_2_ incubator. The PMA-containing culture medium was then replaced with fresh culture medium, and the cells underwent a rest period by incubation for another 72 h.

### 2.7 A549/dTHP-1 co-cultures

Co-cultures of A549 and dTHP-1 cells were prepared in a 12-well plate format. First, 1.5 mL of A549 cells were seeded at a density of 3.9 × 10^4^ cells per well in duplicates and incubated for 72 h to reach confluency. dTHP-1 cells were rinsed with PBS and detached using Accutase (#A6964, Sigma-Aldrich, USA). The detached dTHP-1 cells were then centrifuged at 200 *g* for 5 min and resuspended in fresh culture medium. Following cell counting with a NucleoCounter® NC-200 (ChemoMetec, Denmark), the dTHP-1 cells were diluted to a concentration of 7.8 × 10^3^ cells/mL. The cell culture medium was then aspirated from the wells containing A549 cells, and 1 mL of dTHP-1 cells were seeded on top of the confluent A549 monolayer and left to adhere overnight.

### 2.8 Exposure of A549/dTPH-1 co-cultures to PET nanoplastics

Concentrations of 10 and 100 μg/mL of PET b001 and PET c000 were prepared by vortexing the stock solutions for 15 s prior to dilution in cell culture medium. The culture medium of established A549/dTHP-1 co-cultures were then aspirated, and the cells were exposed to 1 mL of fresh medium containing either PET b001 or PET c000 (10 and 100 μg/mL) or endotoxin-free water (negative control) for 24 h. In experiments measuring cytokines, 1 μg/mL of LPS from *E. coli* (#tlrl-b5lps, Invivogen, France) was included as a positive control to verify the functionality of the assay. The ratio of water to culture medium was kept the same for all the treatments.

### 2.9 Dosimetry of PET nanoplastics in cell culture medium

The sedimentation kinetics of PET-NPs were calculated theoretically and reported in mass per area (µg/cm^2^). In brief, the deposited fraction was calculated by the *in vitro* sedimentation, diffusion and dosimetry (ISDD) model (Hinderliter et al., 2010) assuming a concentration of 100 μg/mL, a diameter of 63.2 nm (values obtained for PET c000 from the analysis of SEM images by ImageJ), and a density of 1.38 g/cm^3^, which is the density of PET. The estimation does not account for a protein corona and assumes no particle aggregation in the CCM. Simulation time was set at 24 h (25 points); dish depth 0.00274 m; volume 1.0 mL; Temperature 310 K; dispersant (CCM) viscosity 0.00096 N s/m^2^, density 1 g/mL (Poon, 2022).

### 2.10 Cell viability

The cell viability of exposed A549/dTHP-1 co-cultures was assessed using alamarBlue® Cell Viability Reagent (#DAL1025, Thermo Fisher Scientific, USA), which measures the intracellular reducing power of living cells. Two 12-well plates were seeded in parallel, and exposures were carried out in duplicate wells on each plate as described above. After 24 h, the medium was aspirated, and the cells were rinsed twice with PBS. In one of the 12-well plates, 1 mL of cell culture medium was added to each well. In the other 12-well plate, 1 mL of 0.2% Triton X-100 (#T8787, Sigma-Aldrich, USA) in culture medium was added to the wells. Both plates were then incubated for 15 min at 37 °C. Five hundred microliters of 30% alamarBlue in RPMI 1640 medium free of phenol red (#11835030, Gibco, Scotland) was then added to the wells of both plates, resulting in a final concentration of 10% alamarBlue. Wells with cell culture medium and alamarBlue but no cells served as blank. The plates were left to incubate for 1 h before transferring 100 μL of the culture media to a black-walled 96-well plate (#94.6000.024, Sarstedt, Germany), using three wells per treatment. Fluorescence 545 nm_ex_/590 nm_em_ was measured using a BioTek Synergy Neo2 multi-mode microplate reader (BioTek Instruments, USA). Following blank subtraction, the mean fluorescent signal for each treatment was calculated. The mean corrected fluorescence of the dead cells was then subtracted from the mean corrected fluorescence of the living cells. Finally, percentage viability was calculated relative to the negative control.

### 2.11 RNA isolation and cDNA synthesis

Total RNA was isolated from cells using Total RNA Purification Plus Kit (#48400, Norgen, Canada). The procedure was performed according to the manufacturer’s protocol, including lysate preparation from adherent cells (Section 1A[i]), genomic DNA removal (Section 2), and total RNA purification (Section 3). As an additional step, the RNA was treated with DNase I on-column during the purification step using RNase-Free DNase I Kit (#25710, Norgen, Canada) to ensure complete removal of residual gDNA. Total RNA concentrations were quantified with a NanoDrop 2000 spectrophotometer (Thermo Fisher Scientific, USA). Conversion of mRNA to cDNA was performed using qScript® cDNA Synthesis Kit (#95047, Quantabio, USA), according to the manufacturer’s instructions, with 1 μg of total RNA as input per 20 μL reaction volume.

### 2.12 Cytokine expression by quantitative polymerase chain reaction (qPCR)

Relative gene expression of *IL1B*, *IL6*, *IL8*, and *TNF* was assessed by quantitative polymerase chain reaction (qPCR). Each reaction was prepared in a 10 μL volume for a 384-well plate format using PerfeCTa SYBR Green FastMix (#95074, Quantabio, USA) in accordance with the manufacturer’s instructions, with 125 nM each of forward and reverse primer (Table 2) and 25 ng of cDNA template. In addition to the samples, a no reverse transcriptase control (NRT) and a no template control (NTC) were included. All samples and controls were run in triplicates for 40 PCR cycles on a QuantStudio 5 Real-Time PCR System (Applied Biosystems, USA), following the manufacturer’s Fast 2-Step Cycling protocol. Gene expression of *IL1B, IL6, IL8,* and *TNF* relative to the negative control was calculated using the 2^−ΔΔCq^ method (Livak and Schmittgen, 2001), with normalization to the geometric mean of the Cq values of three reference genes (*HMBS*, *HPRT1*, and *TFRC*).

**TABLE 2:** Primer sequences used for qPCR.

Gene name	Abbreviation	Fwd primer (5′-3′)	Rev primer (5′-3′)
Interleukin 1 beta	*IL1B*	TGA​GCT​CGC​CAG​TGA​AAT​GA	GGT​GGT​CGG​AGA​TTC​GTA​GC
Interleukin 6	*IL6*	GCA​GAA​AAA​GGC​AAA​GAA​TC	CTACATTTGCCGAAGAGC
Interleukin 8	*IL8*	GTT​TTT​GAA​GAG​GGC​TGA​G	TTT​GCT​TGA​AGT​TTC​ACT​GG
Tumor necrosis factor	*TNF*	AGG​CAG​TCA​GAT​CAT​CTT​C	TTATCTCTCAGCTCCACG
Hydroxymethylbilane synthase	*HMBS*	AGA​AAA​GCC​TGT​TTA​CCA​AG	TTT​TGG​GTG​AAA​GAC​AAC​AG
Hypoxanthine phosphoribosyltransferase 1	*HPRT1*	ATA​AGC​CAG​ACT​TTG​TTG​G	ATA​GGA​CTC​CAG​ATG​TTT​CC
Transferrin receptor	*TFRC*	AAG​ATT​CAG​GTC​AAA​GAC​AG	CTT​ACT​ATA​CGC​CAC​ATA​ACC

### 2.13 Cytokine measurements by enzyme-linked immunosorbent assay (ELISA)

Concentrations of secreted IL-1β, IL-6, IL-8, and TNF proteins were measured in the cell culture supernatant by enzyme-linked immunosorbent assay (ELISA). The following ABTS ELISA Development Kits (PeproTech, UK) were used: Human IL-1 beta (#900-K95, PeproTech), Human IL-6 (#900-K16, PeproTech), Human IL-8 (#900-K18, PeproTech), and Human TNF alpha (#900-K25, PeproTech). ELISA microplates, buffers, and ABTS substrate were purchased separately (#900-K00, PeproTech). Apart from pre-coating the microplate with capture antibodies overnight at 4 °C, the procedure was performed according to the manufacturer’s instructions. Absorbance (OD) was measured at a wavelength of 405 nm with wavelength correction set at 650 nm using a BioTek Synergy Neo2 multi-mode microplate reader (BioTek Instruments, USA), ensuring that the OD did not exceed the recommended maximum for the highest standard concentration. Protein concentrations were interpolated from the standard curve and adjusted for any sample dilution.

### 2.14 Statistics and data presentation

Data were analyzed in GraphPad Prism version 10.4.0 (GraphPad Software, USA) using one-way ANOVA followed by Dunnett’s *post hoc* test to compare each exposure to the negative control. Statistical significance was defined as a P-value of less than 0.05 (adjusted P < 0.05). The positive control was excluded from statistical analysis, as it was included solely to verify assay functionality and was not relevant to the tested hypothesis. Concentrations of IL-6 protein below the limit of detection (LOD) were imputed as LOD/√2 (Hornung and Reed, 1990). The mean ± standard deviation (SD) of three independent experiments is presented if not stated otherwise.

## 3 Results

### 3.1 Particle characterization and dosimetry

**FIGURE 1:**
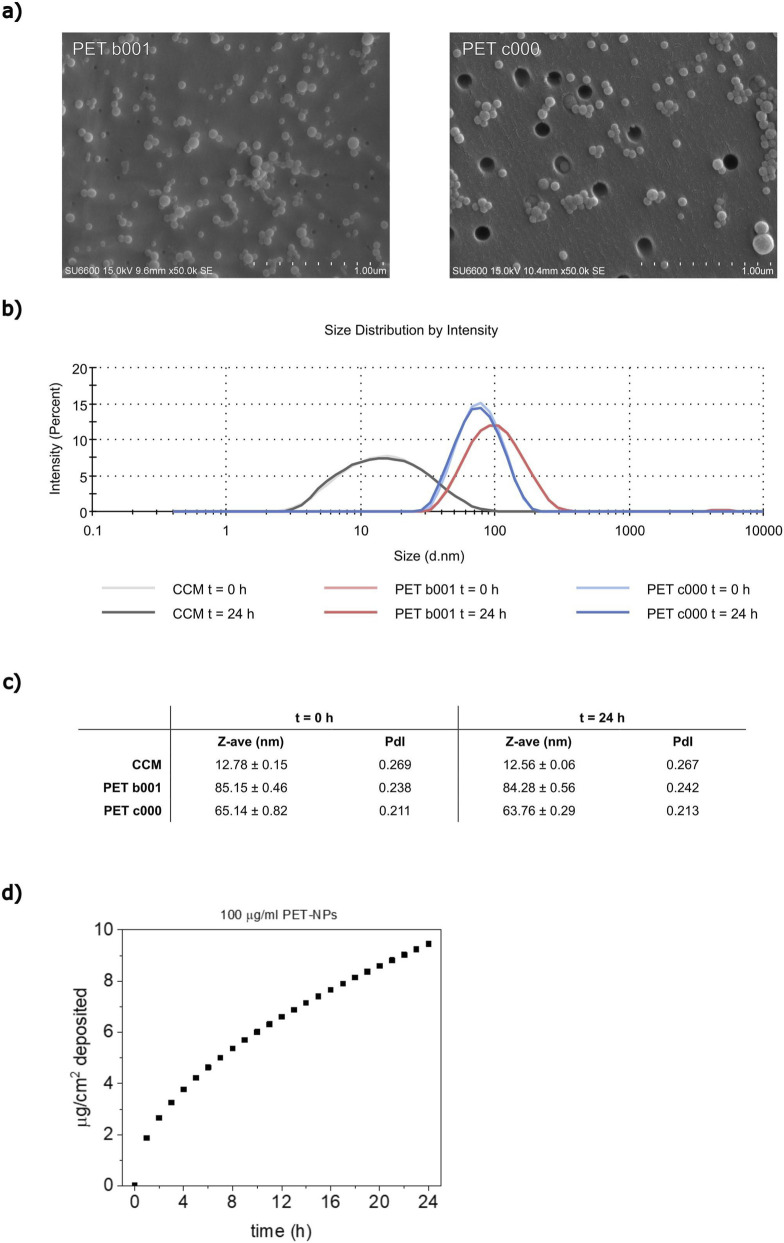
Particle characteristics and dosimetry. FE-SEM micrographs showing the PET-NPs as nanospheres **(a)**. The size distribution by intensity **(b)** and the hydrodynamic diameter (Z-ave) and polydispersity index (PdI) **(c)** of PET b001 and PET c000 (100 μg/mL) were measured immediately after dispersion in FBS-containing CCM (t = 0 h) and after 24 h incubation (t = 24 h) by DLS. The sedimentation kinetics of PET-NPs in cell culture medium (CCM) were calculated using the *In vitro* Sedimentation, Diffusion and Dosimetry (ISDD) model **(d)**. Abbreviations: FE-SEM, field emission scanning electron microscopy; FBS; fetal bovine serum, CCM, cell culture medium; DLS, dynamic light scattering.

Micrographs taken with FE-SEM showed that PET b001 and PET c000 particles displayed nanosphere morphology (Figure 1a). DLS showed that the size distribution, hydrodynamic diameter (Z-ave), and polydispersity index (PdI) of both PET b001 and PET c000 remained almost unchanged after incubation for 24 h in CCM (Figures 1b,c). The size and scattering intensity of CCM remained stable, indicating the absence of bacterial growth. Moreover, the scattering intensity was <6% of the PET-NP samples and thus had no significant influence on the hydrodynamic diameter measurements of the PET-NPs. The stable hydrodynamic diameter and PdI over 24 h indicate good colloidal stability of PET b001 and PET c000 in CCM, with no aggregation of particles observed over time. A PdI between 0.2 and 0.3 suggests a moderately polydisperse particle suspension, meaning that the PET-NPs have a moderately narrow size distribution in CCM, as reported in Figure 1c. Estimation of sedimentation kinetics using the ISDD model (Figure d) indicated that after 24 h in CCM, ∼9.4 µg of PET-NPs per cm^2^ have deposited on the cells at the highest concentration tested (100 μg/mL), corresponding to ∼34% of the particles.

### 3.2 Juice bottle-derived PET-NPs activate Toll-like receptors

**FIGURE 2:**
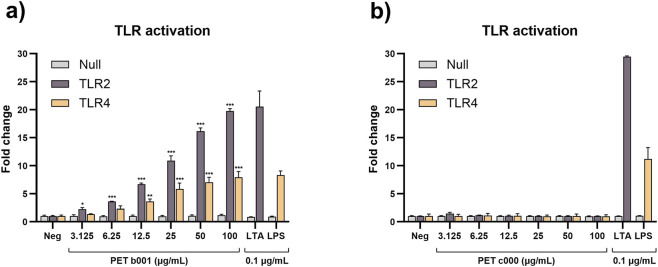
Bar plots showing the activation status of TLR2 and TLR4 in HEK-Blue reporter cells after exposure to PET-NPs. HEK-Blue reporter cells expressing TLR2 or TLR4 were exposed to PET b001 or PET c000 for 24 h, starting at 100 μg/mL and serially diluted two-fold down to 3.125 μg/mL. Endotoxin-free H_2_O served as a negative control, while lipoteichoic acid (LTA) and lipopolysaccharide (LPS) were used as positive controls for TLR2 and TLR4 activation, respectively. Exposure to PET b001 activated TLR2 from the lowest tested concentration (3.125 μg/mL) and TLR4 from 12.5 μg/mL onward **(a)**. In contrast, PET c000 did not activate either TLR2 or TLR4 at any tested concentration **(b)**. No TLR activation was observed in the parental HEK-Blue Null cells following exposure to either particle type **(a,b)**. Statistical significance was assessed using one-way ANOVA with Dunnett’s *post hoc* test comparing each condition to the negative control. *P ≤ 0.05; **P ≤ 0.01; ***P ≤ 0.001. Positive controls (LTA and LPS) were not included in the statistical analysis. Data represent the mean ± SD of three independent experiments. Abbreviations: TLR, Toll-like receptor; LTA, lipoteichoic acid; LPS, lipopolysaccharide.

The presence of microbial components in PET b001 and PET c000 samples were assessed using HEK-Blue reporter cells expressing either TLR2 or TLR4 and NF-κB/AP-1 inducible SEAP. Exposure to PET b001 resulted in significant activation of both TLR2 and TLR4, indicating the presence of microbial components among these particles (Figure 2a). In contrast, neither TLR2 nor TLR4 were activated following exposure to PET c000, indicating the absence of microbial ligands for these receptors (Figure 2b). Parental HEK-Blue Null cells, carrying the same NF-κB/AP-1-inducible SEAP reporter gene but lacking TLRs, showed no increased SEAP activity following exposure to PET b001 or PET c000 (Figures 2a,b). This excludes the possibility that PET b001 enhances SEAP activity in HEK-Blue TLR2 and TLR4 cells via a mechanism independent of TLR activation.

### 3.3 Exposure to PET-NPs does not affect cell viability

**FIGURE 3:**
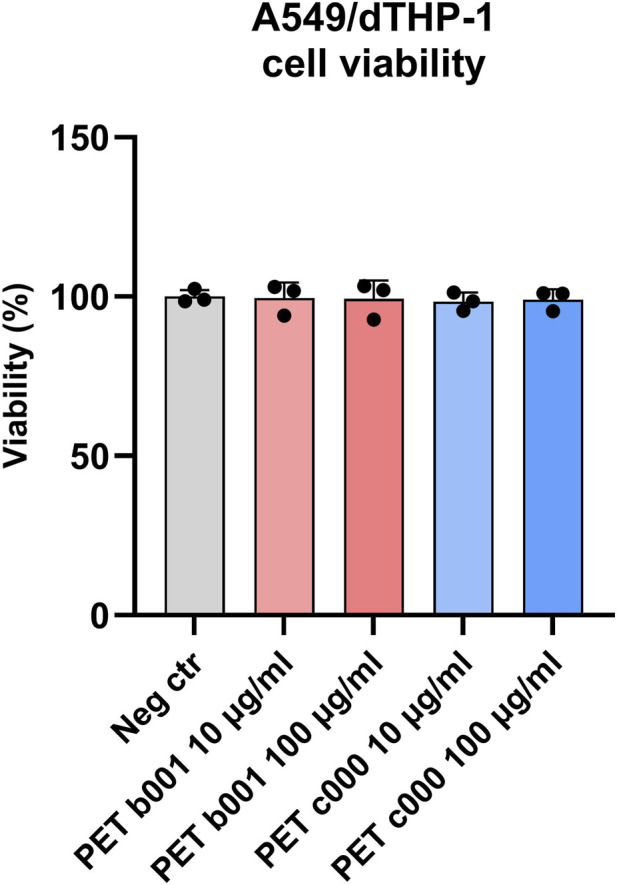
Bar plot showing the cell viability of A549/dTHP-1 co-cultures following exposure to PET-NPs. A549/dTHP-1 co-cultures were exposed to PET b001 or PET c000 (10 or 100 μg/mL), or to endotoxin-free H_2_O as a negative control for 24 h. Cell viability was assessed using alamarBlue. No statistically significant differences were found using one-way ANOVA (P > 0.05). Data represents the mean ± SD of three independent experiments.

A reduction in cell viability would indicate cytotoxic effects of the PET-NPs. Furthermore, as changes in viability may alter the number of cytokine-producing cells, it was important to assess this parameter prior to cytokine analysis to avoid potential confounding effects. The viability of A549/dTHP-1 cells was therefore evaluated following exposure to PET b001 or PET c000 (10 or 100 μg/mL) (Figure 3). This was achieved using the alamarBlue assay, which measures the metabolic activity of viable cells by quantifying the reduction of resazurin to the fluorescent product, resorufin. Given that the A549/dTHP-1 co-cultures had reached confluence prior to exposure, a decrease in fluorescence is more likely due to cytotoxic effects of the PET-NPs than to inhibited cell proliferation. As can be seen in Figure 3, neither PET b001 nor PET c000 caused a reduction in cell viability.

### 3.4 Exposure to PET-NPs triggers pro-inflammatory responses

**FIGURE 4:**
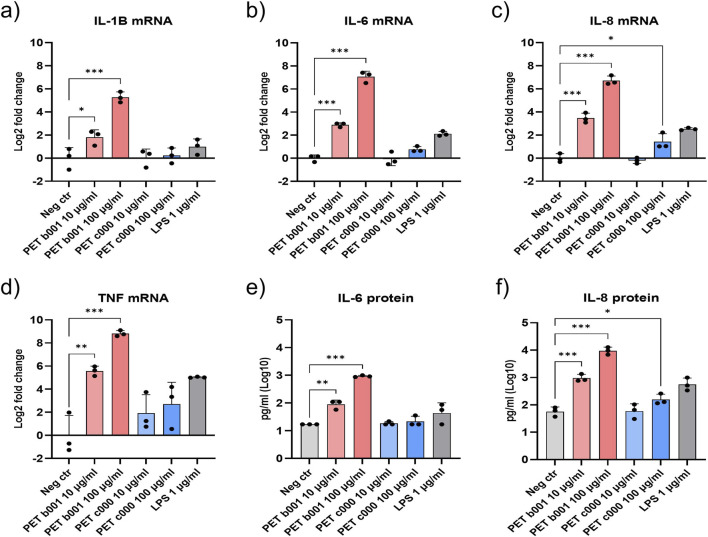
Bar plots showing the gene and protein expression of pro-inflammatory cytokines in A549/dTHP-1 co-cultures following exposure to PET-NPs. IL-1B, IL-6, IL-8, and TNF were analyzed following exposure of A549/dTHP-1 co-cultures to PET b001 or PET c000 for 24 h. Endotoxin-free H_2_O served as a negative control, while LPS served as a positive control. PET b001 (10 and 100 μg/mL) increased mRNA levels of IL-1B **(a)**, IL-6 **(b)**, IL-8 **(c)**, and TNF **(d)** and protein secretion of IL-6 **(e)** and IL-8 **(f)**. The highest concentration of PET c000 (100 μg/mL) increased IL-8 mRNA **(c)** and IL-8 protein secretion **(f)**. Statistical significance was assessed using one-way ANOVA with Dunnett’s *post hoc* test comparing each condition to the negative control. *P ≤ 0.05; **P ≤ 0.01; ***P ≤ 0.001. The positive control (LPS) was not included in the statistical analysis. Since IL-6 protein secretion in the negative control was below the limit of detection (LOD; 24 pg/mL), a value of LOD/√2 was assigned to the negative control for statistical analysis. Data represents the mean ± SD of three independent experiments.

The expression levels of four representative cytokines (IL-1β, IL-6, IL-8, and TNF) were quantified to evaluate the pro-inflammatory potential of PET-NPs in A549/dTHP-1 co-cultures following exposure for 24 h (Figure 4). These cytokines are key mediators of inflammatory signaling in the acute phase of pulmonary inflammation. Following exposure of A549/dTHP-1 co-cultures to PET b001 or PET c000 for 24 h, gene expression was assessed by RT-qPCR and protein secretion was measured by ELISA. Exposure to PET b001 significantly upregulated mRNA expression of all four cytokines (Figures 4a-d) and increased IL-6 and IL-8 protein secretion (Figures e,f), and this was observed at both tested particle concentrations. In contrast, PET c000 showed a more modest response, selectively increasing IL-8 at the highest particle concentration, with elevations in both mRNA expression and protein secretion (Figures 4c,f). Secreted IL-1β and TNF protein levels were below the detection limit for all experimental conditions (data not shown).

## 4 Discussion

In this study, we investigated the cytotoxic and pro-inflammatory potential of PET-NPs synthesized from two different sources using A549/dTHP-1 co-cultures as a model of the alveolar epithelium. PET-NPs were generated either from a post-consumer juice bottle (PET b001) or from commodity PET pellets (PET c000). Particles derived from the juice bottle exhibited microbial contamination, as evidenced by activation of TLR2 and TLR4, whereas particles generated from the commodity PET pellets were contaminant-free.

We did not find evidence of cytotoxicity after acute exposure to any of the PET-NPs, as cell viability remained unaffected. Studies assessing pulmonary cytotoxicity following exposure to PET-NPs remain scarce, and comparable data are therefore limited. To our knowledge, the few available studies have primarily used submerged monocultures. One study tested different concentrations of spherical PET-NPs (0.1–196.79 μg/mL) and observed a modest reduction in viability of A549 cells following 24 h exposure to concentrations of 98.4 μg/mL or higher (Zhang et al., 2022). This concentration is comparable to our highest administered dose of 100 μg/mL, with the same exposure duration, although their PET-NPs were larger than ours (mean size 164 nm versus ∼65 nm). Variations in experimental conditions, such as the use of mono-versus co-cultures, different particle sizes and deposited dose, cell confluency, and the type of viability assay employed, may explain the different findings.

Our results are, however, in line with three other studies. In the first study, human nasal epithelial cells (HNEpC) were exposed to irregularly shaped PET-NPs (1–100 μg/mL) sanded from PET water bottles, and no changes in viability were observed after 24 h (Annangi et al., 2023). By labeling the same PET-NPs with a fluorescent dye in a follow-up study, the authors demonstrated that the viability of murine alveolar macrophages (MH-S) remained unaffected after particle uptake (Tavakolpournegari et al., 2024). While no cytotoxicity was observed in these two studies, other functional changes were found, including loss of mitochondrial membrane potential and an increase of reactive oxygen species (ROS) (Annangi et al., 2023; Tavakolpournegari et al., 2024). Increased ROS production and DNA damage have also been reported for A549 cells following exposure to PET-NPs from ground food containers (Alzaben et al., 2023). In the third study using bottle-derived PET-NPs, no cytotoxic effects were observed following 24-h exposure of human bronchial epithelial cells (BEAS-2B) at concentrations up to 200 μg/mL (Gutiérrez-García et al., 2025).

PET c000 at 100 μg/mL selectively upregulated expression and secretion of IL-8. IL-8 is a chemoattractant for neutrophils and is secreted by alveolar macrophages and epithelial cells as part of the acute inflammatory response to harmful stimuli (Kunkel et al., 1991). A study on various components commonly found in particulate matter (PM), including ultrafine carbon black, ZnCl_2_, FeSO_4_, 1-nitropyrene, LPS, and crystalline silica, compared the gene expression of a large number of cytokines after exposing bronchial epithelial cells (Ovrevik et al., 2009). The authors found that IL-8 was the only gene consistently induced by all components and was, on average, the most highly upregulated cytokine, including in response to sampled PM. Therefore, the increased IL-8 observed following exposure to PET c000 may not be directly related to the plastic polymer itself but could instead reflect a general cellular response to PM, irrespective of its physicochemical properties.

The induced IL-8 response by PET c000 was only evident at the highest administered dose of 100 μg/mL. This dose is likely very high compared to real-life exposures, especially given the low mass-to-number ratio of NPs, although it is commonly used in toxicity studies of MNPs *in vitro* (Schröter and Ventura, 2022). In comparison, the average plastic concentration in human blood samples (n = 22) was 1.6 μg/mL, with one sample exceeding 12 μg/mL (Leslie et al., 2022). To our knowledge, no such data exists for human lung tissue. However, not all the PET-NPs will be in direct contact with the cells, especially due to their nanoscale size which will keep them in suspension for longer. While we do not have experimental data on the delivered PET-NP doses, we estimated the particle sedimentation rate in CCM *in silico* using the ISDD model (Hinderliter et al., 2010). After 24 h, the estimated deposited fraction equals approximately 9.4 μg/cm^2^ or 32.9 µg per well. Most other studies that have exposed submerged cells to NPs do not report the deposited dose, making it difficult to directly compare findings. Nonetheless, our deposited dose may help assess the inherent toxicity of PET-NPs and serve as a representation of a worst-case exposure scenario.

The markedly stronger cytokine response induced by PET b001 compared with PET c000, characterized by increased mRNA levels of IL-1B, IL-6, IL-8, and TNF, along with enhanced secretion of IL-6 and IL-8, is likely attributable to microbial contaminants. This interpretation is supported by the activation of TLR2 and TLR4 by PET b001 in the HEK-Blue hTLR reporter cell assay. We believe that this reflects contamination with components of microorganisms already present in the post-consumer juice bottle, since both the bottle and the PET pellets underwent identical procedures to synthesize PET-NPs. TLRs are key components of the innate immune system and respond to conserved pathogen-associated molecular patterns (PAMPs) by initiating transcription of pro-inflammatory cytokines through NF-κB and MAPK signaling (Kawai et al., 2024). Both TLR2 and TLR4 are present in human lungs and play a crucial role in regulating inflammation triggered by inhaled pathogens (Lafferty et al., 2010). While expressed lower than in alveolar immune cells, functional TLR2 and TLR4 have also been found in human primary alveolar type II epithelial cells (Armstrong et al., 2004). With regards to the A549/dTHP-1 co-culture, both cell types express TLR2 and TLR4 (Kim et al., 2022; Qadri et al., 2018; Silva Lagos et al., 2022; Homma et al., 2004; Regueiro et al., 2009), although A549 cells require addition of soluble CD14, present in serum, for functional TLR4 signaling (Schulz et al., 2002; Thorley et al., 2011). We have previously shown that microbial ligands capable of TLR activation are common in MNP test materials (Haugen et al., 2025). Recognizing such contamination is critical, as it may affect biological outcomes and lead to inaccurate conclusions and contradictory findings between studies.

Several studies have shown significantly higher particle-induced cytokine expression in co-cultures of epithelial cells and macrophages, including A549/dTHP-1 co-cultures, than in corresponding monocultures (Friesen et al., 2022; Tao and Kobzik, 2002; Wottrich et al., 2004). Crosstalk between these cells appears to enhance cellular sensitivity and strengthen the pro-inflammatory response, although the exact mechanism seems unclear. This has also been observed for IL-8, where the authors suggest that TNF secreted by macrophages potentiates IL-8 secretion from epithelial cells, although TNF was not measured (Friesen et al., 2022). Indeed, this mechanism is supported by a study on particulate matter where conditioned media from exposed macrophages in combination with TNF-neutralizing antibodies significantly reduced the IL-8 secretion by A549 cells (Jiménez et al., 2002). However, we did not observe increased TNF secretion, even when TNF mRNA levels were significantly elevated, as seen following exposure to PET b001. While we can only speculate, it is possible that secreted TNF had peaked earlier and subsequently fell below the detection limit due to receptor-mediated internalization, enzymatic degradation, or other clearance mechanisms.

Finally, we would like to address some limitations of our study. Our synthetized PET-NPs are spherical, whereas real-world NPs are expected to exhibit a wider range of morphologies. Furthermore, although microbial contamination likely reflects a realistic exposure scenario, other environmentally relevant particle characteristics, such as adsorption of other pollutants, residual additives, and weathering-induced changes in surface chemistry, were not considered in this study. Moreover, the relevance of the tested particle concentrations to real-world exposure is uncertain, as quantitative data on human exposure to NPs remain limited due to current analytical constraints. Accordingly, these concentrations are best interpreted within a hazard-identification and mechanistic framework. In addition, submerged exposure does not accurately reflect the alveolar lumen and may induce protein corona formation through interactions with CCM components, thereby altering particle surface chemistry and cellular interactions (Abdelkhaliq et al., 2018; Tan et al., 2020). To better mimic physiological conditions of the respiratory tract, cells can be exposed to particles at an air-liquid interface (ALI) (Lacroix et al., 2018). Exposure of A549/dTHP-1 co-cultures to PM2.5 in submerged conditions elicited stronger pro-inflammatory responses than ALI exposure (Wang et al., 2020), whereas others have observed the opposite for poorly soluble nanomaterials (Loret et al., 2016; Lenz et al., 2013). Direct comparisons of exposure conditions are therefore needed when studying pulmonary toxicity of NPs. Although not comparing exposure systems, one recent study measured IL-8 secretion by bronchial cells following ALI-exposure to three different NPs and found no differences (Gosselink et al., 2024). Finally, our study focused exclusively on acute cellular responses to PET-NP exposure. Prolonged and/or repeated exposure, as well as earlier time points, may result in different biological outcomes. The above-mentioned factors should be carefully considered when interpreting our findings.

## 5 Conclusion

In summary, we found that PET-NPs synthesized from a post-consumer juice bottle (PET b001) activated TLR2 and TLR4, indicating the presence of microbial components capable of inducing immune responses. Exposure to these particles increased IL-1B, IL-6, IL-8, and TNF mRNA levels, as well as elevated protein secretion of IL-6 and IL-8 in co-cultured human alveolar epithelial cells (A549) and macrophages (dTHP-1). In contrast, pristine PET-NPs (PET c000) only elicited a modest response at high concentrations by selectively increasing IL-8 mRNA levels and protein secretion. No changes in cell viability were observed for either particle type, consistent with previous studies reporting no cytotoxic effects from MNP exposure. Importantly, our results reveal that microbial components play a critical role in the inflammatory potential of PET-NPs, suggesting that environmental factors significantly influence their toxicity. Future studies on biological effects of NPs are warranted, particularly in occupational health settings, where attention should be directed at NPs that may be contaminated by microbial components.

## Data Availability

The original contributions presented in the study are included in the article/supplementary material, further inquiries can be directed to the corresponding author.
